# Investigation of Hexagonal Mesoporous Silica-Supported Composites for Trace Moisture Adsorption

**DOI:** 10.1186/s11671-015-1159-x

**Published:** 2015-11-17

**Authors:** Li Li, Nian Tang, Yaxue Wang, Wanglai Cen, Jie Liu, Yongyan Zhou

**Affiliations:** Electric Power Research Institute of Guangdong Power Grid Company Limited, Guangzhou, 510080 People’s Republic of China; College of Resources and Environment, Chengdu University of Information Technology, Chengdu, 610225 People’s Republic of China; Institute of New Energy and Low-Carbon Technology, Sichuan University, Chengdu, 610065 People’s Republic of China

**Keywords:** Moisture adsorption, PEI impregnation, HMS, Hydrogen bond

## Abstract

Moisture control is an important part of effective maintenance program for gas-insulated switchgear (GIS). Herein, hexagonal mesoporous silica (HMS) materials were synthesized by adopting dodecylamine as a structure directing agent, which was then employed as a host for supporting polyethylenimine (PEI) without further calcinations or extraction treatment. The physicochemical properties of the silica support and composites were characterized, and the moisture adsorption capacity of these composites was determined. The reserved template agents resulted in a dramatic improvement in moisture adsorption amount. Among them, 50PEI/DHMS showed the highest adsorption value. The enhanced adsorption could be attributed to the generated hydrogen bonding between amino groups and H_2_O molecules and the improved diffusion of moisture into the bulk networks of PEI polymers due to its better spatial dispersion imposed by the long alkyl chains of template agents, which was confirmed by thermogravimetry results and hydrogen efficiency analysis. Moreover, the maintained terminal amino groups of templates could also function as active sites for moisture adsorption. The results herein imply that the PEI/DHMS composites could be appealing materials for capturing moisture in GIS.

## Background

Sulfur hexafluoride (SF_6_), showing extreme inertness characteristics and excellent chemical stability, has been widely used as an electrical-insulating medium in gas-insulated switchgears (GIS) [1, 2]. However, moisture in GIS, particularly in the liquid phase, seriously affects its dielectric withstand strength. Thus, it shall be maintained under a level so that it does not condense into liquid at any expected operating temperatures. Additionally, when the partial discharge occurs caused by the internal insulation defects, SF_6_ would decompose into SF_5_, SF_4_, SF_3_, SF_2_, S_2_F_10_, and other low-fluoride sulfides [3, 4]. And the excessive moisture in the enclosed equipment could accelerate the further reaction of these decomposition products to corrosive gases like SO_2_, SO_2_F_2_, and HF. Hence, to avoid the potential to endanger the safe operation of equipment, a lot of efforts have been made to detect and control moisture content in SF_6_ gas [5–7].

Various kinds of adsorbents (such as Al_2_O_3_, 4A, 5A, 13X zeolites) are located in the insulation chamber as desiccants [3, 8, 9]. Zeolites, as a typical kind of physicosorptive adsorbent, are molecular sieves with regular three-dimensional framework structures, large internal surface area, and excellent thermal stability [10, 11]. The adsorption process appears to be highly influenced by these structural characteristics especially under low pressure, no matter any kinds of adsorbates. The ideal designed materials should adsorb water moisture at a rather low concentration efficiently. However, there are few open literatures about this point as far as we know.

Since its first report in 1992, mesoporous silica has found extensive utility in adsorption, catalysis, and medication due to their high internal surface areas and large pore volumes [12–16]. Compared with the highly oriented mesoporous materials (MCM-41, SBA-15, etc.), hexagonal mesoporous silica (HMS) is a kind of 3-D channel mesoporous silica materials with a disordered structure. HMS can be easily synthesized by a sol-gel process using a primary alkylamine as a structure directing agent at room temperature. The weak hydrogen bonding between silicate precursors and neutral amines would generate during this procedure [16, 17]. Finally, the neutral template in the as-synthesized HMS could be removed facilely by solvent extraction. Chen et al. [18] had employed HMS as a host for supporting polyamines as a CO_2_ capturing agent.

In this paper, polyethylenimine (PEI) modified as-synthesized HMS (without further thermal treatment or solvent extraction) were prepared based on the following two points: (i) The abundant amino groups either from PEI molecules or from the primary amines of template agent would generate hydrogen bond affinity to moisture, which is stronger than van der Waals (physical adsorption) and would significantly improve the adsorption capacity. (ii) The maintained long chains of alkylamines may modify the dispersion state of loaded PEI, which make more active sites exposed and decrease the diffusion resistance for H_2_O to the active sites.

## Methods

Dodecylamine (DDA), tetraethoxysilane (TEOS), and all the other chemicals were purchased from Sinopharm Chemical Reagent Co., Ltd and were utilized without further treatment. Solution was prepared by adding redistilled water (unless explicitly stated).

The as-synthesized HMS was prepared according to the previous reports [18]. Under vigorous stirring at 50 °C, TEOS was added to a solution of DDA in ethanol and distilled water according to the molar ratio of *n*_TEOS_:*n*_ODA_:*n*_CH3CH2OH_:*n*_H2O_ = 1.00:0.27:6.50:36.00. Then the substrate mixture was aged at ambient temperature overnight. After that, the resulting precipitate was filtered and dried at 105 °C. The obtained white powder was denoted as as-synthesized DHMS.

PEI was incorporated into the pores of DHMS through wet impregnation, which has been detailed covered in our previous report [19]. Two grams of support powders was added into methane solution (40 ml) of PEI, then the mixture was stirred for 2~3 h in a sealed glass vessel and another 6~10 h in fume hood to allow methane evaporation. The residue was then held at 100 °C overnight under reduced pressure. According to the loading contents of PEI $\left(a\%=\frac{\mathrm{The}\ \mathrm{additive}\ \mathrm{weight}\ \mathrm{of}\ \mathrm{P}\mathrm{E}\mathrm{I}}{\mathrm{The}\ \mathrm{aggregated}\ \mathrm{weight}\ \mathrm{of}\ \mathrm{P}\mathrm{E}\mathrm{I}\ \mathrm{and}\ \mathrm{DHMS}}\right)$, the modified DHMS composites were denoted as αPEI/DHMS.

Nitrogen adsorption-desorption isotherms were obtained at 77 K under relative pressures ranging from 0.005 to 0.99 using TristarII3020 (V1.03) surface area and porosity measurement system (Micromeritics Inc., USA). The thermal stability of modified mesoporous silica was determined using thermogravimetric (TG) analysis (NETZSCH STA 409 Luxx, Selb/Bavaria, Germany). About 10 mg of samples was heated from room temperature to 600 °C at a heating rate of 10 K/min in air atmosphere. Elemental analysis was performed on Flash EA1112 (Thermo Finnigan, USA). Fourier transform infrared spectrometer (FTIR; IR Affinity-1, SHIMADZU, Japan) was employed to record the IR spectrograms of samples. Potassium bromide (KBr) plates mixed with 1/50 of sample were made by applying 20 t of oil pressure and then were scanned from 400 to 4000 cm^−1^ with resolution of 0.2 cm^−1^.

**Fig. 1:**
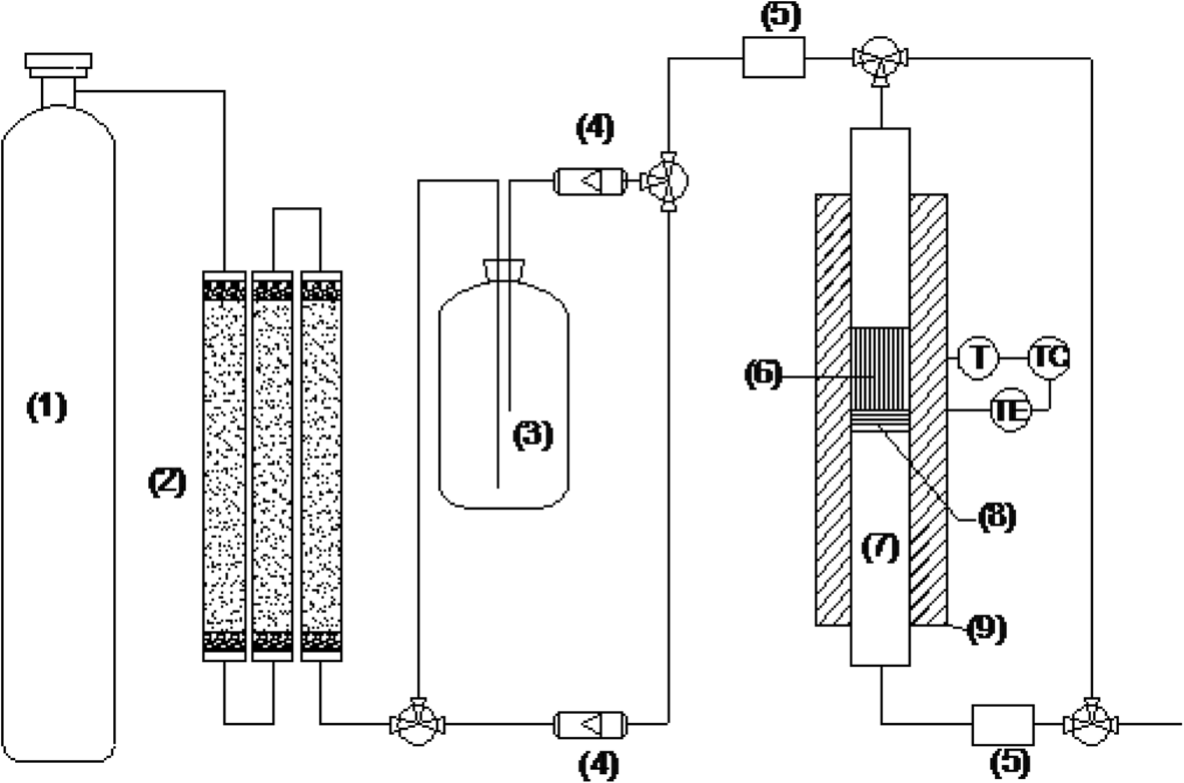
Schematic diagram of the experimental setup: *(1)* compressed gas cylinder, *(2)* pretreatment system, *(3)* concentrated sulfuric acid, *(4)* flow controllers, *(5)* dew point sensors, *(6)* packed particles, *(7)* reaction tube, *(8)* silica wool support, *(9)* insulation, *T* for thermocouple, *TC* for temperature controller, and *TE* for heating element

The kinetic-equilibrium tests of moisture adsorption were carried on a quartz fixed-bed (i.d. = 8 mm and L = 200 mm) equipped with a controlled temperature programming furnace, as it was described in our previous work [19] and some improvement had been made in this section (shown in Fig. 1). Adsorbent particles (40–60 mesh) were preheated at 105 °C for a span of 6 h with purge flow at 50 ml/min under atmospheric pressure. Then the inflow gas was switched in after cooling down to room temperature until the pseudo-equilibrium reached. It should be noted that both the nitrogen inflow gases were dried deeply over the pretreatment system loaded with anhydrous calcium chloride and by flowing through concentrated H_2_SO_4_. The H_2_O concentrations in the inlet and outlet flow were recorded in real time by dew point sensors (CS-iTEC, CS220), respectively.

The moisture adsorption capacity (expressed as *q*_e_ here, mg/g) could be calculated by integrating the obtained breakthrough curves, which was fitted as Eq. (1):

1 $$ {q}_{\mathrm{e}}=\frac{1}{m}{\displaystyle {\int}_0^tk\left({Q}_{\mathrm{in}}{C}_{\mathrm{in}}-{Q}_{\mathrm{e}\mathrm{ff}}{C}_{\mathrm{e}\mathrm{ff}}\right)\mathrm{d}\mathrm{t}.} $$

where *m* is the weight of adsorbent particles (g), *k* the apparent density of effluent gas (mg/ml), *Q*_in_ is the inlet flow rate (ml/min) and *Q*_eff_ is the effluent flow rate (ml/min), *C*_eff_ is the effluent H_2_O concentration (ppm), and *t* is the used time (min) until *C*_eff_ reaches *C*_in_. After that, purge flow was switched in again at 105 °C for another 6 h until the adsorbed water was almost regenerated.

## Results and discussion

**Fig. 2:**
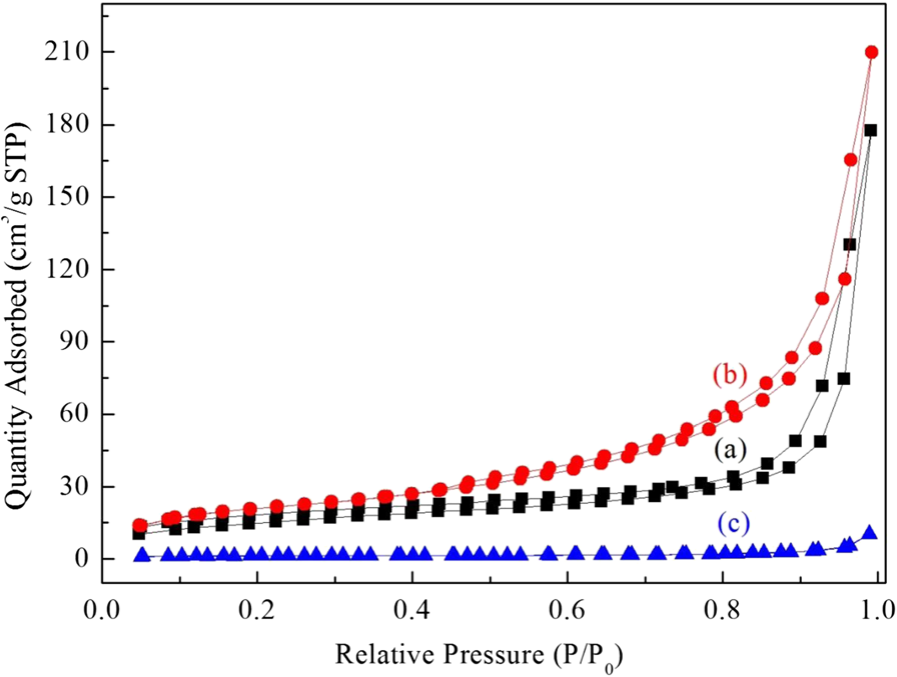
Nitrogen adsorption-desorption isotherms: *(a)* as-synthesized DHMS, *(b)* 20PEI/DHMS, and *(c)* 50PEI/DHMS

The N_2_ adsorption-desorption isotherms and the structural characteristics of the as-synthesized DHMS and PEI/DHMS were presented in Fig. 2 and Table 1, respectively. These samples showed the typical type IV isotherms according to the IUPAC classification, indicating their mesoporous character. And the appearance of H1 hysteresis loop was associated with porous materials with uniform shapes and narrow pore size distributions, which is in accordance with the previous literatures [20, 21]. Note that the BET surface area and total pore volume of as-synthesized DHMS were dramatically reduced to 54.15 m^2^/g and 0.27 cm^3^/g in comparison to the calcined sample [18, 22]. It could be attributed that the surfactant species filled in the channels took up some accessible volume for nitrogen molecules.

**Table 1:** Textural characteristics of DHMS before and after PEI loading

Samples	SSAs^a^ (m^2^/g)	Vtotal^b^ (cm^3^/g)	Pore size^c^ (nm)
HMS-C [18]	952	0.82	–
DHMS	54.15	0.27	20.29
20PEI/DHMS	75.28	0.32	17.25
40PEI/DHMS	13.48	0.14	15.93
50PEI/DHMS	4.44	0.02	14.43

^a^Specific surface areas were calculated using the Brunauer-Emmett-Teller (BET) method based on the isotherm linear plot (*P*/*P*_0_ = 0.06~0.20)

^b^Vtotal was determined by the single point liquid nitrogen adsorption capacity at a relative pressure of 0.99

^c^Pore size means the adsorption average pore width

As it was discussed [23], the structural characteristics of supports provided accommodation for impregnated polyamines and played a critical role in determining the theoretical maximum loading content. In our results, the SSAs and pore volume fell gradually with PEI loading, and when the PEI loading was high to 50 %, the pore volume was extremely small, only 0.02 cm^3^/g left.

It should be considered that apart from the physical characteristics, the chemical nature of supports also had significant impact on the nature of amine adsorption sites. Heydari-Gorji et al. [24] had reported that PEI supported on pore-expanded MCM-41 whose surface was covered with a layer of long alkyl chains was found to be more efficient for CO_2_ adsorption than PEI supported on the corresponding calcined silica. They deduced the layer of surface alkyl chains that played an important role in enhancing the dispersion of PEI and decreasing the diffusion resistance. Kuwahara et al. [25] also supported that the interaction between primary amines and the surface acid sites on the silicate may stabilize and/or change the structure of loaded PEI and potentially enhanced the accessibility of the rest of the amines to incoming adsorbates. Thereout in this paper, it was possible that the un-extracted directing agent improved the spatial configuration of PEI in the pores of supports. Yet, this speculation would be further confirmed in the following results.

**Fig. 3:**
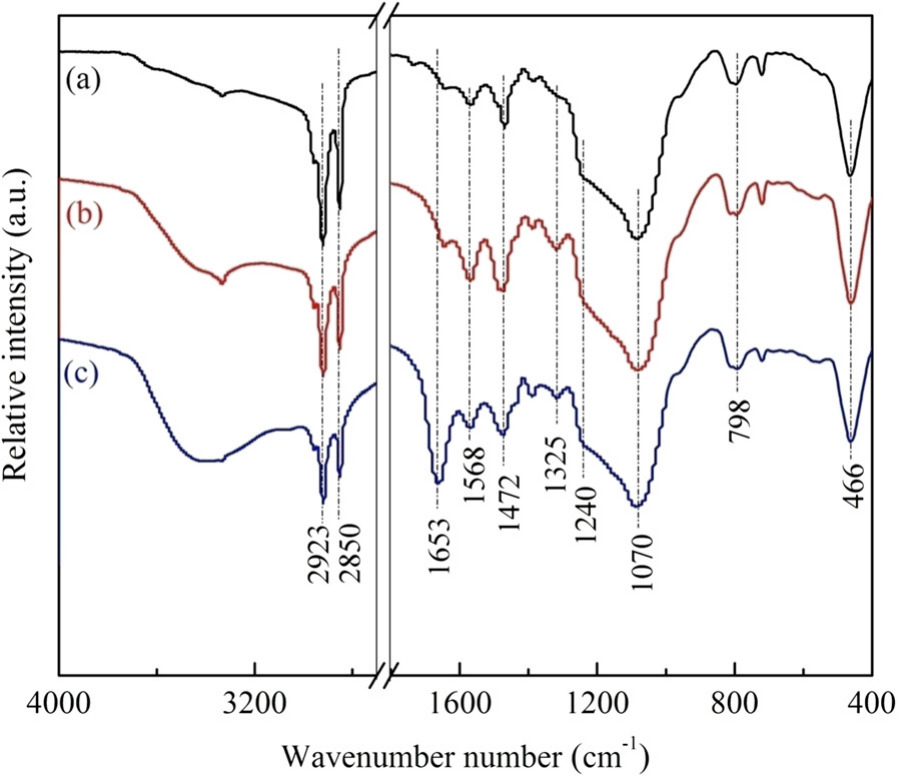
FTIR spectrum of *(a)* as-synthesized DHMS, *(b)* 20PEI/DHMS, and *(c)* 40PEI/DHMS

FTIR spectra in the range of 4000–400 cm^−1^ were used to confirm the functionalization of the as-synthesized DHMS with polyamines, which was illustrated in Fig. 3. The broad adsorption band around 3440 cm^−1^ was attributed to the stretching vibration of Si–OH groups with absorbed water molecules and also the –NH_2_ group present in the template agent [20]. The distinct peaks at 1240, 1070, and 466 cm^−1^ corresponded to the asymmetric/symmetric stretching, and bending modes of ≡Si–O–Si≡, coupled with the observed band at 798 cm^−1^ assigned to the asymmetric/symmetric stretching vibration of tetrahedral SiO_4_ structural units, confirmed its structural matrix of DHMS [15, 20, 26]. Meanwhile, there emerged several characteristic peaks for surfactant dodecylamine, like the bands at 2923 and 2850 cm^−1^ caused by the asymmetric and symmetric C–H stretching motion of CH_3_ groups, and the peak at 1472 cm^−1^ attributed to the stretching vibration of CH_2_ group [22, 27].

It was worth noting that the vibration band due to the bending mode of NH_2_ shifted to 1568 cm^−1^ in this result from 1593 cm^−1^ in pure dodecylamine, which might be caused by the formation of hydrogen bond between the hydrogen atoms of NH_2_ groups and the oxygen atoms of silica tetrahedron [28]. Furthermore, a rather small band could be observed at l653 cm^−1^ assigned to the bending motion of NH_3_^+^ group, implying the existence of terminal alkylamine in its protonated formation.

Additionally, the hydrophilic amino would inevitably adsorb some water moisture or CO_2_ from its surroundings, which accounted for the increased growth of the peaks at 1653 and 1325 cm^−1^ (due to the weakly adsorbed CO_2_) after PEI loading.

**Fig. 4:**
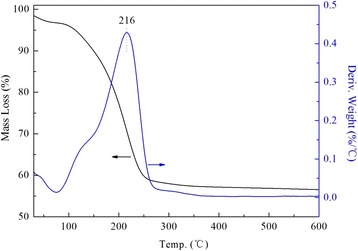
TG and DTG profiles of DHMS

TG study was carried out to determine the thermal stability of loaded PEI. As it was presented in Fig. 4, the weight loss of DHMS could be divided into three stages [29]. The weight loss below 150 °C was assigned to the physical desorption of water and other volatile species. The main weight loss in the second range from 150 to 300 °C could be attributed to the decomposition and combustion of the organic templates in the sieve apertures. The maximum weight loss rate occurred at 216 °C rounded to the boiling points of dodecylamine (248 °C), similar to the report [30]. The weight loss in the third temperature range up to 500 °C was due to the dehydroxylation of the surface silanol groups.

**Fig. 5:**
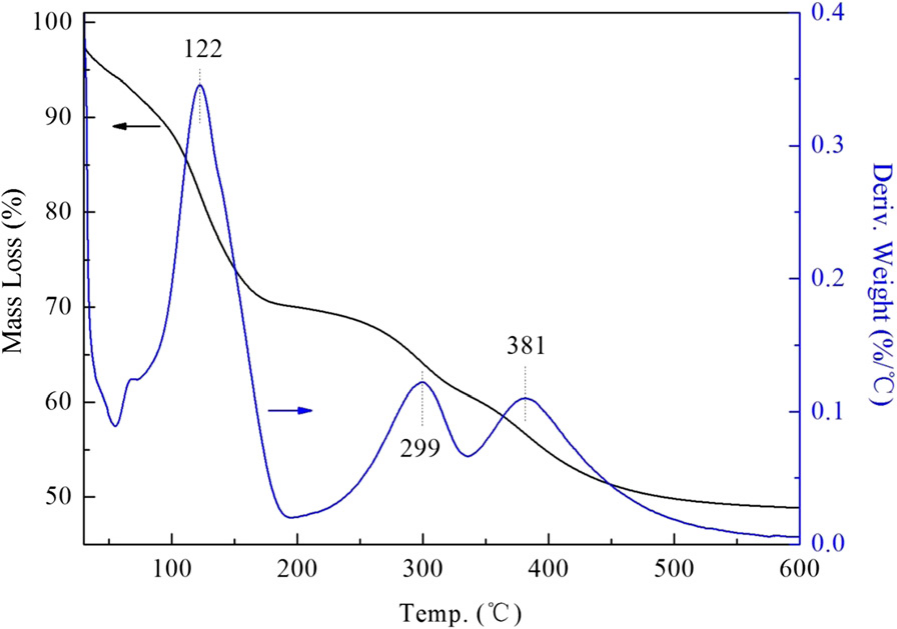
TG and DTG profiles of 20PEI/DHMS

However, the PEI-modified DHMS showed entirely different ups and downs. With consideration of the following two reasons, it was believed that the first sharp peak at 122 °C for 20PEI/DHMS (Fig. 5) was related to the partial decomposition of loaded PEI: (i) in the temperature range up to 150 °C, pure PEI hardly showed any mass loss [31] while it was about 10 % for DHMS mainly caused by the releasing of physically adsorbed air moisture. It seems like that there were other transform processes. (ii) The previous works have deduced that better dispersion of PEI with higher volatility in nano-porous supports would lead to the decomposition of polymers at a lower temperature. As such, the decrease of PEI decomposition temperature could be ascribed to its well dispersion by the template agents, which confirmed the BET results. In addition, the two peaks at 299 and 381 °C were reasonably associated with the decomposition of residue PEI.

**Fig. 6:**
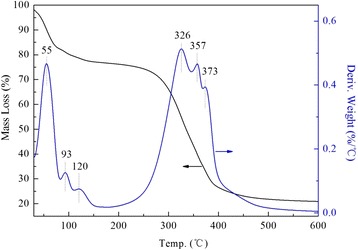
TG and DTG profiles of 50PEI/DHMS

When the loading amount exceeded the maximum capacity, the pores may essentially be blocked completely, and a part of loaded PEI would form a polymeric film on the external surface of supports, leading to the re-rising decomposition temperature (Fig. 6). And that was why the mass loss in this stage was maintained at 25 %.

**Fig. 7:**
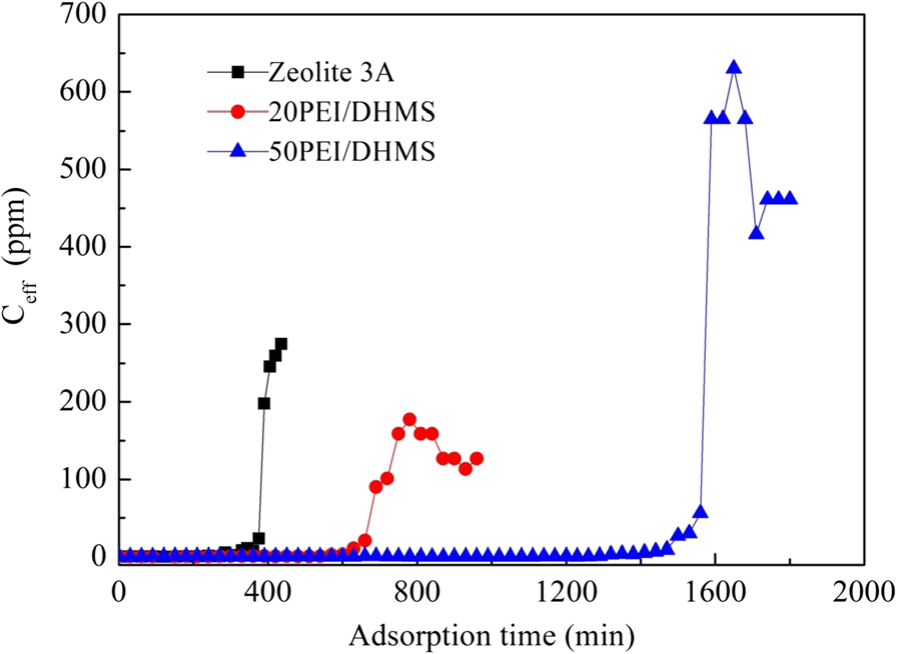
Breakthrough curves obtained for trace moisture adsorption on zeolite 3A, 20PEI/DHMS and 50PEI/DHMS under atmospheric pressure at ambient temperature

Breakthrough curves for 20PEI/DHMS and 50PEI/DHMS under a moisture/nitrogen atmosphere were displayed in Fig. 7. By contrast, H_2_O adsorption performance on zeolite 3A was also carried out. And the calculated adsorption capacities for these samples were listed in Table 2. High adsorption capacity was observed at high PEI loading. A plausible explanation was that the abundant amino groups either from the existing template agents or the introduced polyamines could function as active sites for moisture capture. In this process, the hydrogen atoms in amino groups, whose constraint to electron was weakened by nitrogen atoms, showed some affinity to the oxygen atoms in H_2_O molecules. In addition, the interaction between the nitrogen atom from an adjacent amino group and the hydrogen atom of the H_2_O molecule could also be established. That is, the “density” of active sites (the strength of reaction) would also influence the moisture adsorption properties. This could explain the obtained moisture adsorption capacity was higher over 50PEI/DHMS than 20PEI/DHMS. Actually, this type of interaction had broad application in selective adsorption and separation [32–34].

**Table 2:** Moisture adsorption properties of 20PEI/DHMS and 50PEI/DHMS

Samples	H_2_O adsorption capacity (mmol/g)	H content in adsorbents (mmol/g)	Hydrogen efficiency (mol_H2O_/mol_H_)
20PEI/DHMS	1.23	49.61	0.02
50PEI/DHMS	10.10	77.13	0.13
Zeolite 3A	0.79	–	–

Due to its hydrophobic property, the moisture adsorption capacity on DHMS was not carried out on DHMS

As it was reported, zeolite 3A is a synthetic crystalline potassium aluminosilicate that is usually obtained by ion exchange from the sodium form of zeolite type A (known as zeolite 4A) [8, 35]. Within its cages, the adsorbed water molecules also hydrogen bonded with each other associated with the K^+^ ions. Hefti et al. [36] had also reported a steep increase in the water adsorption equilibrium isotherm due to the high affinity of H_2_O towards zeolite 13X. But it should be noted that the amount of amino groups in PEI-modified composites were much more extensive than the K^+^ ion in zeolite 3A, and that was why the PEI-modified composite adsorbents had better adsorption properties than zeolite 3A and the moisture adsorption capacity significantly increased with the increase of PEI loading.

Here, hydrogen efficiency was defined as the number of adsorbed H_2_O molecules for each nitrogen atom in composite adsorbents. It was always hypothesized [23] that a higher loading amount which aggregated spontaneously in channels would generate greater diffusion resistance for adsorbate molecules to the active sites in the inner layer of PEI, therefore resulting in lower hydrogen efficiency and long tails for adsorption curves. However, the hydrogen efficiency was enhanced to 0.13 mol_H2O_/mol_H_ for 50PEI/DHMS (see Table 2), even though the impregnated PEI almost stuffed the available pores of supports completely. Moreover, both the adsorption curves of these samples showed steep increases in the saturation stage for the adsorption equilibrium capacities, indicative of efficient access of H_2_O molecules into the adsorption sites and conversely a diffusion restriction of H_2_O into the composites. Although more data might be gathered to be ascertained (the pore structure may also play an important role) [18], the dramatic improvement in kinetic adsorption of H_2_O might highly possibly be related to the presentation of productive surfactants within the mesopores of supports, which significantly improved the dispersion state of loaded PEI. Better dispersion of the polymers in the pores would result in better accessibility of the amine active sites and lower diffusion resistance for H_2_O molecules to the active sites in the inner layers of PEI.

It should also be noted that a decrease in adsorption capacity over amine-modified composites was observed in the long/cyclic operation under continuous flow mainly due to the leaching of the physisorbed PEI from the channels of support and also its deactivation at high temperatures [19, 37]. However, neither these problems would occur when the modified composites were located under static condition at constant temperature (room temperature) for moisture adsorption, even though the dynamic adsorption tests were proceed in this paper to investigate its adsorption properties.

## Conclusions

In this work, novel PEI-modified HMS was synthesized for trace moisture removal through the hydrogen bonding. The uniform mesoporous channels of HMS could be preserved after polyamine loading, but the specific surface area and pore volume would descend significantly. The kinetic moisture adsorption on these modified composites showed a dramatic improvement in adsorption capacity and hydrogen efficiency. For instance, the moisture adsorption amount of 50PEI/DHMS was about eight times as much as that of 20PEI/DHMS. This enhancement in adsorption capacity was attributed to the enhanced H_2_O diffusion into deeper polyamines layers and more exposed moisture affinity sites. TG results further confirmed by that the decomposition of PEI became easier on the supports with un-extracted template agents. It was deduced that the extended alkyl chains spatially dispersed the loaded PEI molecules. Furthermore, the terminal amino groups could also function as active sites for moisture capture. Hydrogen bonding was formed between amino groups and H_2_O molecules, which also played a crucial role in improving moisture adsorption properties.
